# Ion Beam Assisted E-Beam Deposited TiN Microelectrodes—Applied to Neuronal Cell Culture Medium Evaluation

**DOI:** 10.3389/fnins.2018.00882

**Published:** 2018-12-04

**Authors:** Tomi Ryynänen, Maria Toivanen, Turkka Salminen, Laura Ylä-Outinen, Susanna Narkilahti, Jukka Lekkala

**Affiliations:** ^1^BioMediTech Institute and Faculty of Biomedical Sciences and Engineering, Tampere University of Technology, Tampere, Finland; ^2^NeuroGroup, BioMediTech Institute and Faculty of Medicine and Life Sciences, University of Tampere, Tampere, Finland; ^3^Laboratory of Photonics, Tampere University of Technology, Tampere, Finland

**Keywords:** titanium nitride, microelectrode array, MEA, IBAD, cell culture medium

## Abstract

Microelectrode material and cell culture medium have significant roles in the signal-to-noise ratio and cell well-being in *in vitro* electrophysiological studies. Here, we report an ion beam assisted e-beam deposition (IBAD) based process as an alternative titanium nitride (TiN) deposition method for sputtering in the fabrication of state-of-the-art TiN microelectrode arrays (MEAs). The effects of evaporation and nitrogen flow rates were evaluated while developing the IBAD TiN deposition process. Moreover, the produced IBAD TiN microelectrodes were characterized by impedance, charge transfer capacity (CTC) and noise measurements for electrical properties, AFM and SEM for topological imaging, and EDS for material composition. The impedance (at 1 kHz) of brand new 30 μm IBAD TiN microelectrodes was found to be double but still below 100 kΩ compared with commercial reference MEAs with sputtered TiN microelectrodes of the same size. On the contrary, the noise level of IBAD TiN MEAs was lower compared with that of commercial sputtered TiN MEAs in equal conditions. In CTC IBAD TiN electrodes (3.3 mC/cm^2^) also outperformed the sputtered counterparts (2.0 mC/cm^2^). To verify the suitability of IBAD TiN microelectrodes for cell measurements, human pluripotent stem cell (hPSC)-derived neuronal networks were cultured on IBAD TiN MEAs and commercial sputtered TiN MEAs in two different media: neural differentiation medium (NDM) and BrainPhys (BPH). The effect of cell culture media to hPSC derived neuronal networks was evaluated to gain more stable and more active networks. Higher spontaneous activity levels were measured from the neuronal networks cultured in BPH compared with those in NDM in both MEA types. However, BPH caused more problems in cell survival in long-term cultures by inducing neuronal network retraction and clump formation after 1–2 weeks. In addition, BPH was found to corrode the Si_3_N_4_ insulator layer more than NDM medium. The developed IBAD TiN process gives MEA manufacturers more choices to choose which method to use to deposit TiN electrodes and the medium evaluation results remind that not only electrode material but also insulator layer and cell culturing medium have crucial role in successful long term MEA measurements.

## Introduction

A microelectrode array (MEA) is a common tool to measure the electrical activity of various cell types *in vitro* and to provide an electrical stimulus to the objects under study. The applications of MEAs vary from basic biological research to drug screening and toxicity testing. In neuroscience, it has been found to be applicable for *in vitro* drug screening and toxicity testing (Johnstone et al., 2010; Ylä-Outinen et al., 2010). Recently, the rise of human pluripotent stem cell (hPSC)-based technologies for human cell-based modeling, including disease modeling, has benefitted from MEA technology (Falk et al., 2016; Odawara et al., 2016).

In its simplest form, MEA consists of a glass substrate, a metal layer containing electrodes, tracks, and contact pads, and an insulator layer with openings on the electrodes and the contact pads. Even though metal electrodes such as Pt, Au, or Ti can be used, they have limitations in their performance. For this reason, metallic microelectrodes are usually coated with a porous material that increases the effective surface area ratio (SAR) and decreases the impedance, leading to a higher signal-to-noise ratio of the electrodes (Bauerdick et al., 2003). Since the early days of MEA (Thomas et al., 1972), platinum black (Pt black) has been one of the most commonly used coating materials for low impedance electrodes. It has excellent electrical characteristics, but in addition to obvious cost issues, a major drawback is that Pt black has been reported to have problems with mechanical stability during long-term use (Heim et al., 2012). Iridium oxide (IrOx), even as a rather common *in vivo* electrode material (Cogan, 2008), has not reached notable popularity for *in vitro* microelectrodes. This is likely to be at least partly due to its tendency to lose the low impedance state rather rapidly in a liquid environment (Gawad et al., 2009). Carbon nanotube-based solutions do exist (Gabay et al., 2007; Samba et al., 2014), and even though excellent performance has been reported, they are still more a topic of academic interest than a real choice for active use. The only commonly used substitute for Pt black has been titanium nitride (TiN) (Janders et al., 1996), especially in commercial solutions. Depending on the deposition parameters and methods, the morphology of a TiN thin film may vary a lot from plain to highly columnar. The latter is seen as increased SAR and decreased impedance. Although, some doubts about the performance of TiN exist (Weiland et al., 2002), it can generally be considered as the least problematic high-performance microelectrode coating developed to date. In addition to *in vitro* electrodes, TiN can be used also in *in vivo* applications (Stelzle et al., 2001).

**Figure 1:**
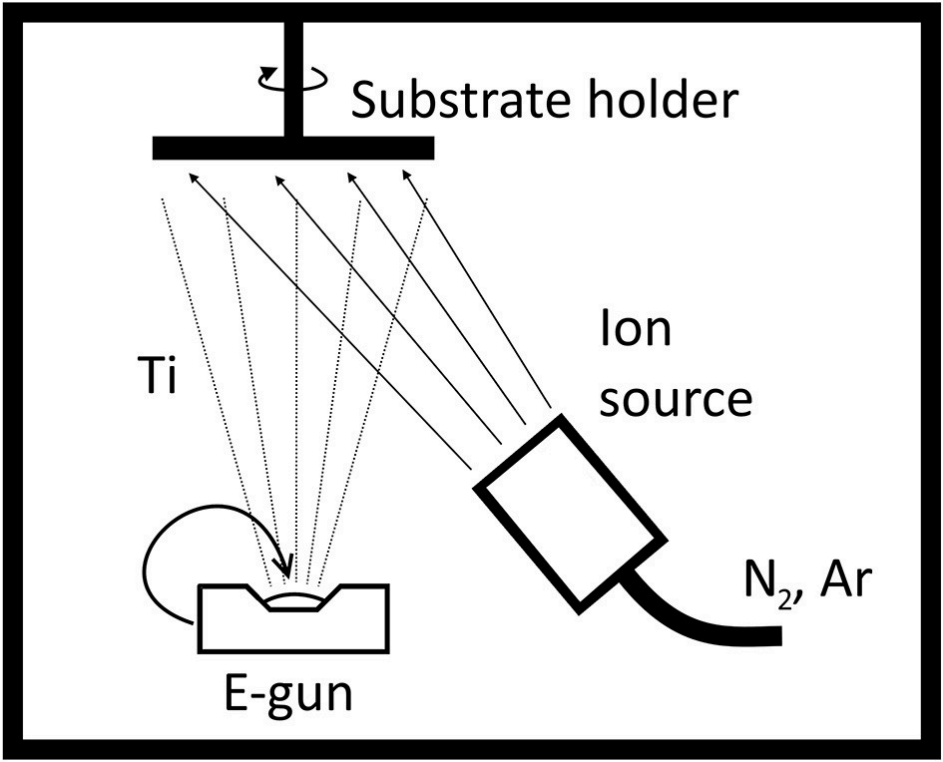
Schematic of an ion beam assisted deposition system. Briefly, e-beam evaporated titanium is bombarded with nitrogen and argon ions to form a TiN thin film on substrates in a vacuum chamber.

There exists a wide range of methods for the fabrication of TiN coatings. Because TiN is applied as the last layer on MEAs in the fabrication process, finding an etching process that is not harmful for the MEA insulator layer, typically Si_3_N_4_, and underlying track material, commonly titanium, might be challenging and may require additional process steps for preparing the etch mask. Thus, a lift-off process is favored with TiN. Because photoresist is needed for lift-off and the melting temperature of the glass substrate set limits for the maximum allowed temperature during the TiN deposition process, certain common TiN deposition processes such as atomic layer deposition (ALD) (Xie et al., 2014), thermal chemical vapor deposition (CVD) (Wagner et al., 2008), and physical vapor deposition (PVD) (Gahlin et al., 1995; Peng et al., 2015) techniques must be ruled out when selecting the TiN deposition method for the MEAs. For this reason, reactive sputtering has been the only method used to deposit TiN on MEA electrodes (Egert et al., 1998; Cyster et al., 2002; Bauerdick et al., 2003; Gabay et al., 2007). However, there is also an alternative method for the low temperature deposition of TiN: ion beam assisted deposition (IBAD) in which the e-beam evaporated titanium is bombarded by a flux of low energy nitrogen and argon ions from the ion source to form TiN (Figure 1). The dominating mechanism in TiN formation is adsorption of ambient gaseous atoms on the growth surface (Hubler et al., 1988). Over the last three decades, several groups have reported their IBAD TiN experiments for hard coatings (Guzman et al., 1998; López et al., 2001) and more general materials science (Hubler et al., 1988; Huang et al., 2000; Yokota et al., 2004) applicable not only on hard coatings but also, for example, on decoration coatings and microelectronics diffusion barriers. By contrast, as far as we know, IBAD TiN has not been applied on MEAs previously.

In this paper, we evaluated different deposition parameters, including evaporation and nitrogen flow rates, for optimal IBAD TiN microelectrode coating. The coatings are characterized by impedance, charge transfer capacity (CTC) and noise measurements for electrical properties, AFM and SEM for topological imaging, and EDS for material composition. Comparison to sputtered TiN electrodes of commercial MEAs (Multi Channel Systems MCS GmbH) is also reported. To verify the biocompatibility and performance of these novel IBAD TiN microelectrodes, we cultured and measured hPSC-derived neuronal networks for 3 weeks. The neuronal networks were grown in two different cell culture media: neural differentiation medium (NDM) (Heikkilä et al., 2009) and BrainPhys (BPH)-supplemented medium recently introduced by Bardy et al. (2015) to evaluate possible medium derived effects on MEA grown cultures.

## Materials and Methods

### IBAD TiN Deposition Process Development

Microscope slides (76 mm × 26 mm × 1 mm; Gerhard Menzel GmbH) were used as substrates while optimizing the IBAD TiN deposition parameters. The slides were cleaned with acetone and isopropanol in an ultrasound bath, rinsed with DI water and dried with a nitrogen blow. Cleaned slides were placed in an Orion BC-3000 series box coater (System Control Technologies) equipped with a Telemark 246 e-beam source, Saintech Series III ST55 gridless ion source, Saintech ion current density monitor, and a Meissner trap for 100 nm IBAD TiN depositions. Table 1 includes different parameter values tested during process development. In all depositions, a filament current of ~20 A was used, and the vacuum during deposition was in the 10^−5^ Torr range (~10^−3^ Pa). The substrate holder was rotated at 5 rpm during the deposition. The 99.995% purity Ti pellets used in both IBAD TiN depositions and later in Ti track depositions in the MEA fabrication were purchased from g-materials. Right after the deposition, the color of the thin films was evaluated visually.

**Table 1:** Deposition parameters tested during IBAD TiN process optimization and AFM characterization results for each sample.

**Sample**	**Set anode voltage [V]**	**N_2_ flow [sccm]**	**Ar flow [sccm]**	**Deposition rate [Å/s]**	**Color**	**Surface area ratio [%]**	**Notes**
1	225	13.2	3.3	1	gold-brown	5.4	
**2**	**225**	**13.2**	**3.3**	**2**	**purple-bronze**	**13.1**	
3	225	13.2	3.3	3.5	light gold	5.1	
4	225	13.2	3.3	5	gray	3.4	
5	225	10.8	1.2	2	brown	4.3	
6	225	9.9	6.6	1	gold	6.0	Unstable deposition
7	140	8.3	8.3	2	gray	4.5	1 AFM measurement only
8	225	16.8	4.2	1	gold	4.4	Ion beam pulsed 8 s ON, 7 s OFF

*Parameters of sample 2 (bolded) were chosen to be used in the IBAD TiN MEA fabrication due to the highest surface area ratio*.

### AFM Measurements

While optimizing the IBAD TiN deposition process, an atomic force microscope (XE-100 AFM, Park Systems) equipped with an ACTA probe (AppNano; radius of curvature, 6 nm) was used for measuring the effective surface area ratio (SAR). An area of 1 μm × 1 μm was measured in intermittent mode for each sample. XEI analysis software (Park Systems) was used for calculating the SAR as per the formula

(1) $$SAR=\frac{A_{S}-A_{G}}{A_{G}}*100$$

where *A*_*G*_ is the plain geometric area and *A*_*S*_ is the total surface area of the corresponding region. The result was finally given as a mean of two areas measured from the same sample. In addition, the software was used to calculate the root-mean-square roughness R_q_.

### IBAD TiN MEA Fabrication

Microscope slide grade glass plates (49 mm × 49 mm × 1 mm; Gerhard Menzel GmbH) were used as substrates for the MEAs. The slides were cleaned with acetone and isopropanol in an ultrasound bath and oxygen plasma before 400 nm of titanium was e-beam deposited at 5 Å/s on the slides. Electrode sites (30 μm in diameter), tracks and contact pads were patterned to the titanium layer in a wet etching [120 H_2_O: 4 H_2_O_2_ (30%): 3 HF (50%)] process in which PR1-2000A positive photoresist (Futurrex, Inc.) was used as an etching mask. Next, 500 nm of silicon nitride was PECVD deposited as an insulator layer at 300°C. PR1-2000A was again used as an etching mask when reactive ion etching with SF_6_ and O_2_ gases was performed with Vision 320 RIE (Advanced Vacuum) to etch the openings on electrode sites and contact pads. The etching mask was not removed after the etching but was reused as a lift-off mask in the IBAD deposition of 400 nm of TiN. For comparison purposes, also MEA versions with 200 nm layer of IBAD TiN as well as MEAs without TiN were fabricated. Just prior to TiN deposition, a 10 min Ar sputter etch was run with the ion source to remove the native oxide layer on titanium electrode sites. IBAD TiN deposition parameters were as follows: anode voltage, 225 V; filament current, 20 A; N_2_ flow, 13.2 sccm; Ar flow, 3.3 sccm; ion current density, 14 μA/cm^2^; ion current density monitor bias, 35.0 V; deposition rate, 2 Å/s; and vacuum, 10^−5^ Torr range (~10^−3^ Pa). The substrate holder was rotated at 5 rpm during the deposition. Finally, lift-off was performed in an acetone ultrasound bath. Either an in-house made PDMS ring or Spikebooster^TM^ 6-well culture chamber (BioMediTech) (Kreutzer et al., 2012) was attached on MEAs to form a pool for Dulbecco's phosphate buffered saline (DPBS) or cell culture media. All of the photolithography masks used in this work were in-house fabricated with a μPG501 direct writing system (Heidelberg Instruments Mikrotechnik GmbH) on chrome mask blanks from Clean Surface Technology Co.

### Impedance and Charge Transfer Capacity Characterization

The pools on the MEAs were filled with DPBS (PBS Dulbecco w/o Ca++, Mg^2+^, Biochrom GmbH), and the MEAs were placed in a temperature chamber at 37°C inside petri dishes for at least 20 h. Subsequently, the MEAs were decreased to room temperature for at least 1 h before the impedance measurement. MEA-IT60 from MCS, a dedicated device for measuring the impedances of all the microelectrodes of a MEA, was used as the measurement device. The measurement was performed at 1 kHz frequency with the sinusoidal test signal being 100 mV and an external Ag/AgCl pellet acting as a grounding electrode. Faulty electrodes existing both in commercial and in-house made MEAs were excluded before calculating mean values for each MEA. For a few randomly selected electrodes of in-house made IBAD TiN MEAs and, for comparison, also of pure Ti MEAs without TiN electrode coating an additional electrochemical analysis was performed. Frequency dependency of the impedance and CTC were measured with Iviumstat potentiostat (Ivium Technologies B.V.). The frequency range was from 1 to 100 kHz and a Pt wire (ALS-Japan) was used as the counter electrode in the impedance measurement. CTC was integrated from the third CV curve when the voltage was ramped between −0.9 and 0.9 V. The same Pt wire acted as the counter electrode as in the impedance measurements, and the reference electrode was DRIREF-2 (World Precision Instruments). Scan rate was 100 mV/s.

### Noise Characterization

Noise characterization was performed as part of the cell culture experiments, where the cell culture medium acted as an electrically conducting solvent. After taking the MEAs from the incubator, they were at first left to stabilize in headstage for 3 min without recording the data. Then, the MEAs were measured for 10 min with the MEA2100 MEA system, MC_Rack software, and temperature controllers TC02 set at 37°C (all from MCS). The voltage signal was filtered (200–3,000 Hz bandpass), and the noise for each electrode of each MEA was calculated as an estimate of the standard deviation of background noise previously described in Quiroga et al. (2004). In calculating noise values for each MEA-medium combination, electrodes with a noise value above 7.0 μV were excluded as they were considered faulty electrodes. Mann-Whitney U-test was performed to indicate statistical significances: for each MEA type between the media at days 6 and 18, and for each medium type between the MEAs at days 6 and 18. *P* < 0.05 were considered significant.

### SEM Imaging and EDS

**Figure 2:**
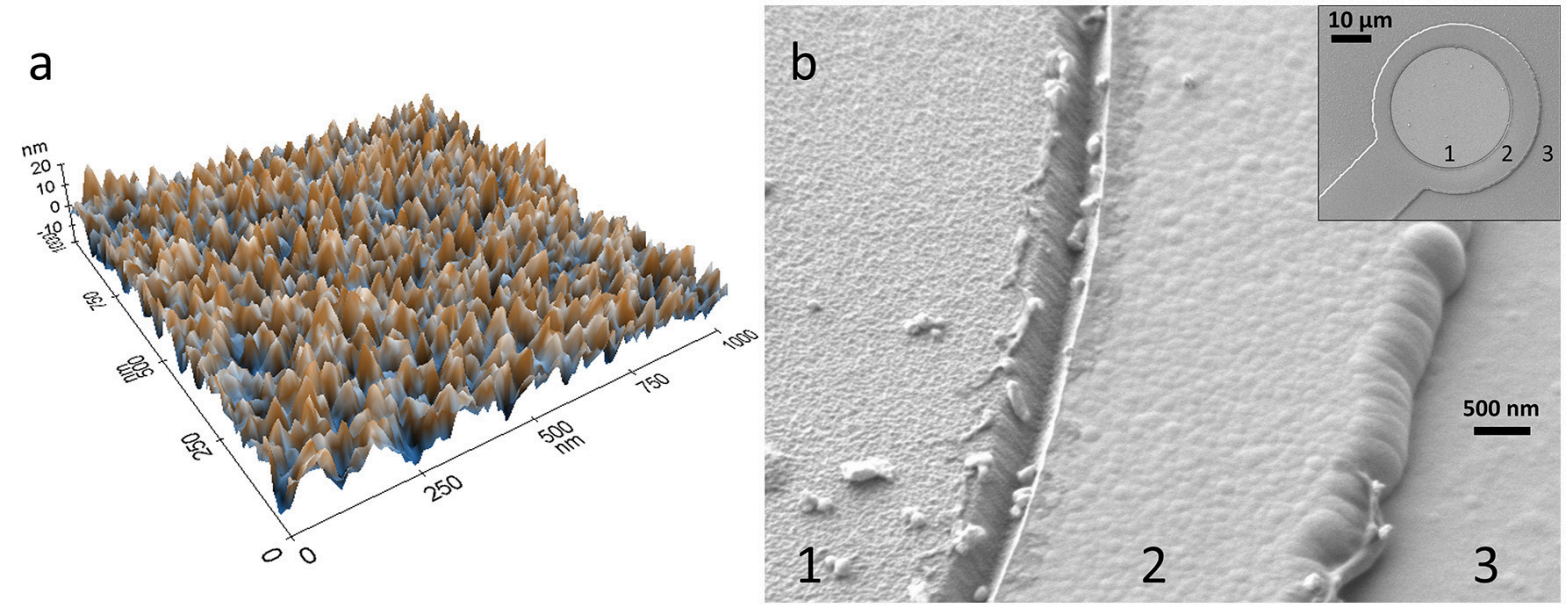
Topological imaging of IBAD TiN. **(a)** AFM image of IBAD TiN surface of sample 2, and **(b)** SEM images of a 30 μm IBAD TiN microelectrode, where 1 is IBAD TiN on Ti, 2 is Si_3_N_4_ on Ti, and 3 is Si_3_N_4_.

Zeiss Crossbeam 540 FIB-SEM (Carl Zeiss Microscopy GmbH) with a Gemini II SEM column and Oxford Instruments X-Max^n^ 80 EDS detector was used in SEM imaging and EDS measurements. In the imaging, the acceleration voltage was 1 kV, and magnifications in Figure 2b were 1.16 and 15.35 kX. In the EDS measurements, acceleration voltages from 7 to 15 kV were used.

### Neural Differentiation

The human embryonic stem cell (hESC) line Regea 08/023 was used in the experiments. BioMediTech has approval from the Finnish Medicines Agency (FIMEA) to perform research with human embryos (Dnro 1426/32/300/05). There are also supportive statements from the regional ethical committee of Pirkanmaa Hospital District for the derivation, culturing, and differentiation of hESCs (R05116). Neurons were differentiated from hESCs as previously described (Lappalainen et al., 2010). Neuronal differentiation medium (NDM) consisted of 1:1 DMEM/F12 and Neurobasal medium supplemented with 1 × B27, 1 × N2, 2 mM GlutaMax (all from Gibco Invitrogen), and 25 μ/mL penicillin/streptomycin (Lonza Group Ltd) and, during the differentiation stage, 20 ng/ml of basic fibroblast growth factor (bFGF) (R&D Systems) as previously described (Lappalainen et al., 2010) with or without low-dose naltrexone LDN193189 (100 nM; Stemcell Technologies, Inc.).

### MEA Preparation and Adherent Culture

MEA preparations were performed as previously described (Heikkilä et al., 2009) with some modifications. MEAs (10 BMT MEAs and 9 60-6wellMEA200/30iR-Ti from MCS) were combined with SpikeBoosters^TM^ (BioMediTech) (Kreutzer et al., 2012) and coated with 0.05% (w/v) polyethylenimine (PEI) incubated overnight, washed with sterile H_2_O, and coated with 20 μg/ml of mouse laminin (both from Sigma-Aldrich) and incubated overnight. A 48-well plate (Thermo Scientific) was coated with 20 μg/ml or 10 μg/ml of mouse laminin in wells with or without coverslips (Ø 9 mm, VWR).

The 8 week pre-differentiated neurospheres were dissected into small cell aggregates (ø~50–200 μm), and 7–10 of them were plated onto the coated MEA wells and the control 48-well plate, which were filled with NDM. During the 1st week, the medium was gradually switched to BrainPhys medium (BPH) consisting of BPH Neuronal Medium supplemented with 1 × NeuroCult SM1 Neuronal Supplement, 1 × N2 Supplement-A (all from Stemcell Technologies), GlutaMax to 2 mM final concentration, and 25 μ/ml penicillin/streptomycin for half of the cells. Additionally, from 2 days in adherent culture 1 mM cyclic adenosine monophosphate (cAMP) and 200 nM ascorbic acid (AA, both from Sigma-Aldrich) were added to the media and from 7 days after plating 8 ng/ml of bFGF and 10 ng/ml of brain-derived neurotrophic factor (BDNF, Gibco Invitrogen) were added to the media. The cells were maintained in a humidified incubator at 37°C and 5% CO_2_, and half of the medium was refreshed 3 times per week. The cells were imaged weekly using a phase contrast microscope (Eclipse Ts2R, Nikon). In addition, the control plate was maintained in Cell-IQ (Chip-Man Technologies) 10 days after plating for 26 h with a 1 h imaging interval (Supplementary Videos 1–3). Spontaneous activities of neuronal networks were measured for 10 min twice per week.

### Immunocytochemistry

The control plate cells were fixed after 12 days in adherent culture, and immunocytochemical staining was performed as previously described (Lappalainen et al., 2010). Primary antibodies, rabbit polyclonal anti-Microtubule-Associated Protein 2 (MAP2) (1:400; Millipore), mouse anti-beta-III Tubulin (β-tub) (1:1000; Sigma-Aldrich), chicken anti-Glial Fibrillary Acidic Protein (GFAP) (1:4000; Abcam), mouse anti-Synaptophysin (1:500; Sigma-Aldrich), chicken MAP2 (1:4000; Novus), and chicken β-tub (1:4000; Abcam) were used together with secondary antibodies Alexa 488 donkey anti-rabbit, Alexa 568 donkey anti-mouse and Alexa 647 goat anti-chicken (all 1:400; Invitrogen). In addition, the nuclei of the cells were stained with 4',6-diamidino-2 phenylindole (DAPI), which was included in the mounting medium (Prolong Gold, Molecular Probes). The cells were imaged with a fluorescence microscope (Olympus IX51, Olympus Corporation) and a laser scanning confocal microscope (LSM 780, Carl Zeiss).

### MEA Signal Analysis and Statistics

Signal analysis from the MEA data was performed using MATLAB (The MathWorks, Inc.) with a custom-made analysis program based on work by Quiroga et al. (2004) in which the spike detection threshold was set to 5, and spikes larger than 500 times the standard deviation of noise were excluded as artifacts. An electrode was regarded as an active electrode if the spike frequency was more than 0.04 Hz. The threshold was determined by measuring spike rates from MEAs without cells and MEAs with TTX-silenced neuronal cultures (data not shown). For spike waveform analysis, 0.8 ms of voltage signal before and 1.76 ms after the largest absolute value of the spike from the filtered data were clipped. The detector dead time between two waveforms was 1.48 ms. Bursts were detected using a MATLAB code based on work by Kapucu et al. (2012) with additional conditions: burst detection was only applied to channels where the total spike frequency was at least 0.167 Hz (10/min). Thereafter, burst analysis criteria included a median of more than two spikes per burst and more than 1 burst per electrode. For each MEA type, Mann-Whitney U-test was performed to indicate statistical significances between the media at each measurement time point. *P* < 0.05 were considered significant.

## Results

### IBAD TiN Process Development

With the assumption that the highest SAR would lead to the lowest microelectrode impedance, we focused on finding the IBAD deposition parameters that would give the highest SAR for the TiN thin film. Briefly, the purpose was to find deposition parameters for a thin film that we expect to give the lowest impedance, not necessarily the purest TiN from material science point of view. The deposition parameters tested while optimizing the IBAD TiN deposition process are presented in Table 1. The effect of changing deposition rates from 1 to 5 Å/s was tested for the same anode voltage and gas flow rate. In addition, some experiments with different anode voltages (sample 7), gas flow rates (samples 5–8) and pulsing of the ion beam (sample 8) were tested. Too high evaporation rate (sample 4) or a too low nitrogen flow rate (sample 7) led to gray thin films resembling pure Ti, which indicates that the conditions did not support the formation of TiN. Lower deposition rate (samples 1 and 2) and higher nitrogen content to argon (sample 5) were seen as brownish thin films, which more closely resembled the almost black thin film in MCS's sputtered TiN MEAs. The remainder (samples 3, 6, and 8) were goldish, which is considered to be the color of TiN hard coatings (Jiang et al., 2004).

In the AFM measurements, purple-bronze-colored sample 2 clearly had the highest SAR of 13.1%. The root-mean-square roughness (R_q_) was 3.1 nm. The AFM image of sample 2 is shown in Figure 2a. According to the assumption of the highest SAR giving the lowest impedance and noise level, we chose the deposition parameters of sample 2 to be used as the deposition parameters in MEA fabrication. Figure 2b shows an SEM image of the IBAD TiN microelectrode. The slight pillar-like structure of TiN can be seen in the image. The EDS measurements showed < 1% variation for the N/Ti ratio, indicating excellent homogeneity of the coating. However, as N is a light element and produces only the Kα peak that partly overlaps with the Ti Lα peak, EDS is better suited for comparative analysis than exact quantification of the N/Ti ratio.

### MEA Performance Characterization

The experimental details are shown in Table 2. Once we had determined the optimal IBAD TiN deposition parameters, we fabricated a batch of IBAD TiN MEAs (hereafter referred to as BMT MEAs) in a 6-well layout mimicking MCS's 60-6wellMEA200/30iR-Ti-w/o array design. In this design, the microelectrodes are grouped in six 3 × 3 electrodes areas for a total of 6 areas with 9 electrodes / MEA. Both in IBAD TiN MEAs and MCS's MEAs the diameter of the electrodes is 30 μm. Table 3 presents the impedance values for the included results, which are grouped by both MEA type and medium used in the cell experiments. Before the cell experiments, the BMT's IBAD TiN electrodes had ~2 × higher impedance compared with that of MCS's sputtered TiN electrodes, approximately 90 kΩ vs. 45 kΩ, respectively. However, as the impedances of Au, Pt or ITO MEAs, i.e., MEAs without a porous electrode coating are typically ~10 × higher (~1 MΩ), the impedance of IBAD TiN is still comparable to sputtered TiN electrodes. After the cell experiments, the impedance of both IBAD and sputtered TiN electrodes increased >100 kΩ; thus, in this sense as well, the behaviors of the two electrode types were comparable. For IBAD TiN MEAs in BPH medium the after cell experiments impedance was significantly lower, only 35 kΩ, but this is because of severe insulator layer corrosion (Figure 7c). Thus, the result is not reliable, as the impedance in this case is not impedance of the electrodes only but rather impedance of both electrodes and tracks. IBAD TiN MEAs in NDM medium, on the contrary, suffered only minor corrosion (Figure 7d), indicating that we can consider their impedance values reliable enough for comparison. The insulator layer of MCS MEAs survived the cell experiments without visible corrosion in both media.

**Table 2:** Experimental setup for MCS MEAs and BMT MEAs.

**MEA type**	**Impedance measurement before use**	**Cell experiments**	**Impedance measurement after use**
MCS MEA	**before E1**	E1^*^, E2^*^, **E3**	**after E2**
BMT MEA	**before E3**	**E3**	**after E3**

*The impedance of each MEA was measured before and after the cell experiments. All the cell experiments (E1–E3) included both NMD and BPH medium tests*.

* *Results excluded due to incubator malfunction*.

*Included measurements are bolded: impedance measurement results for MCS MEAs from before cell experiment E1 and after experiment E2 and for BMT MEA before and after cell experiment E3. Cell experiment results are presented from cell experiment E3*.

**Table 3:** Mean impedance of 30 μm TiN microelectrodes at 1 kHz before and after cell experiments performed in different cell culture media.

			**Before cell experiments**		**After cell experiments**		
**MEA manufacturer**	**TiN type**	**Medium**	**AVG [kΩ]**	**Stdev [kΩ]**	**AVG [kΩ]**	**Stdev [kΩ]**	**Count of MEAs**	Count of cell experiments^**^
BMT	IBAD	NDM	83	5	143	38	5	1
BMT	IBAD	BPH	87	5	35^*^	26^*^	5	1
BMT	IBAD	none	94	21	137	16	2	0
MCS	Sputtered	NDM	46	32	114	86	5	2
MCS	Sputtered	BPH	43	30	101	97	4	2

* *Unreliable result as the Si_3_N_4_ insulator layer was almost completely corroded*.

** *Between impedance measurements*.

**Figure 3:**
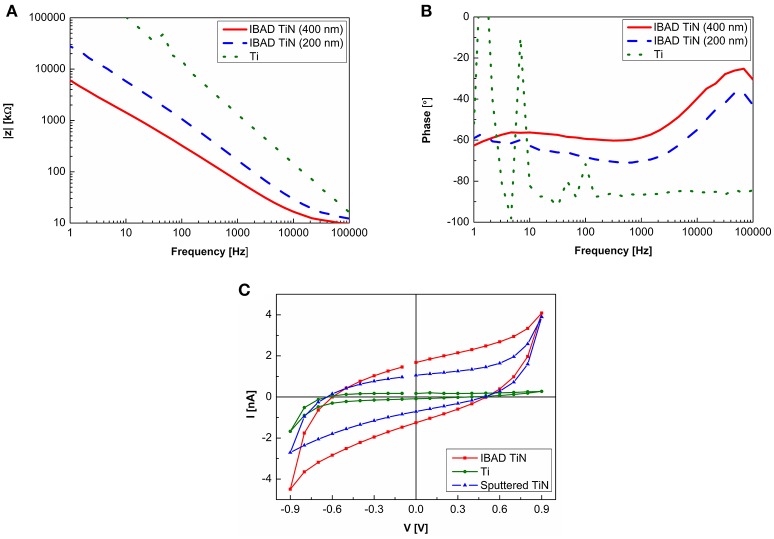
Electrochemical analysis of 30 μm electrodes. **(A)** Magnitude and **(B)** phase of the impedance as a function of frequency of IBAD TiN and Ti electrodes, and **(C)** the third CV curves measured for calculating the charge transfer capacity of IBAD TiN, sputtered TiN, and Ti electrodes.

The measurement of impedance as a function of frequency show (Figures 3A,B) that the thickness of the IBAD TiN strongly affects to the impedance. Decreasing the thickness from 400 to 200 nm about doubles the impedance (at 1 kHz). As expected, compared with the Ti electrodes without the TiN coating the IBAD TiN coating significantly decreases the impedance and also improves the stability at low frequencies. Charge transfer capacity (CTC) integrated from the third CV curves (Figure 3C) was 3.3 ± 0.2 mC/cm^2^ for IBAD TiN microelectrodes and about one tenth of that for Ti electrodes without TiN coating. For MCS MEAs CTC of 2.0 ± 0.2 mC/cm^2^ was measured.

**Figure 4:**
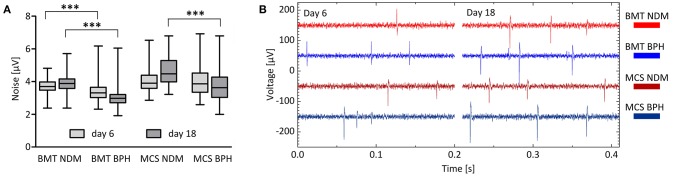
Noise in each MEA type and medium combination. **(A)** Estimation of the standard deviation of background noise of each MEA type and medium combination at two time points. Data are expressed as the median (band inside the box) with interquartile range (IQR; box) and minimum and maximum values (whiskers) at two time points: 6 and 18 days on MEA. BMT, BioMediTech MEA; MCS, Multi Channel Systems MEA; NDM, neuronal differentiation medium; BPH, BrainPhys medium. ***p ≤ 0.001. In addition, the noise level of BMT MEAs was significantly lower from that of MCS's sputtered TiN MEAs under the same time point and condition (*p* < 0.001 for all). **(B)** Examples of raw measurement data of each MEA type and medium combination at two time points. Curves have been shifted vertically for clarity.

The noise level of each MEA type and medium combination was evaluated by calculating the estimate for standard deviation of background noise from 10 min cell measurement data (Quiroga et al., 2004). The results are summarized in Figure 4A. Briefly, the noise level of BMT MEAs was significantly lower from that of MCS's sputtered TiN MEAs under the same time point and condition (*p* < 0.001 for all). Moreover, BPH medium decreased the noise significantly compared with that of the NDM medium (at day 6 in BMT MEAs, *p* ≤ 0.001, and at day 18 in both MEA plate versions, *p* ≤ 0.001). However, typical examples of raw measurement data plotted in Figure 4B show well that, despite the differences in both numerical impedance and noise results, in practice there is no notable difference in the base noise levels and the signal peaks can be separated from the noise equally well with each MEA type and medium combination.

### Effect of Cell Culture Medium

#### Neuronal Network Formation in NDM and BPH Media and BMT and MCS MEAs

**Figure 5:**
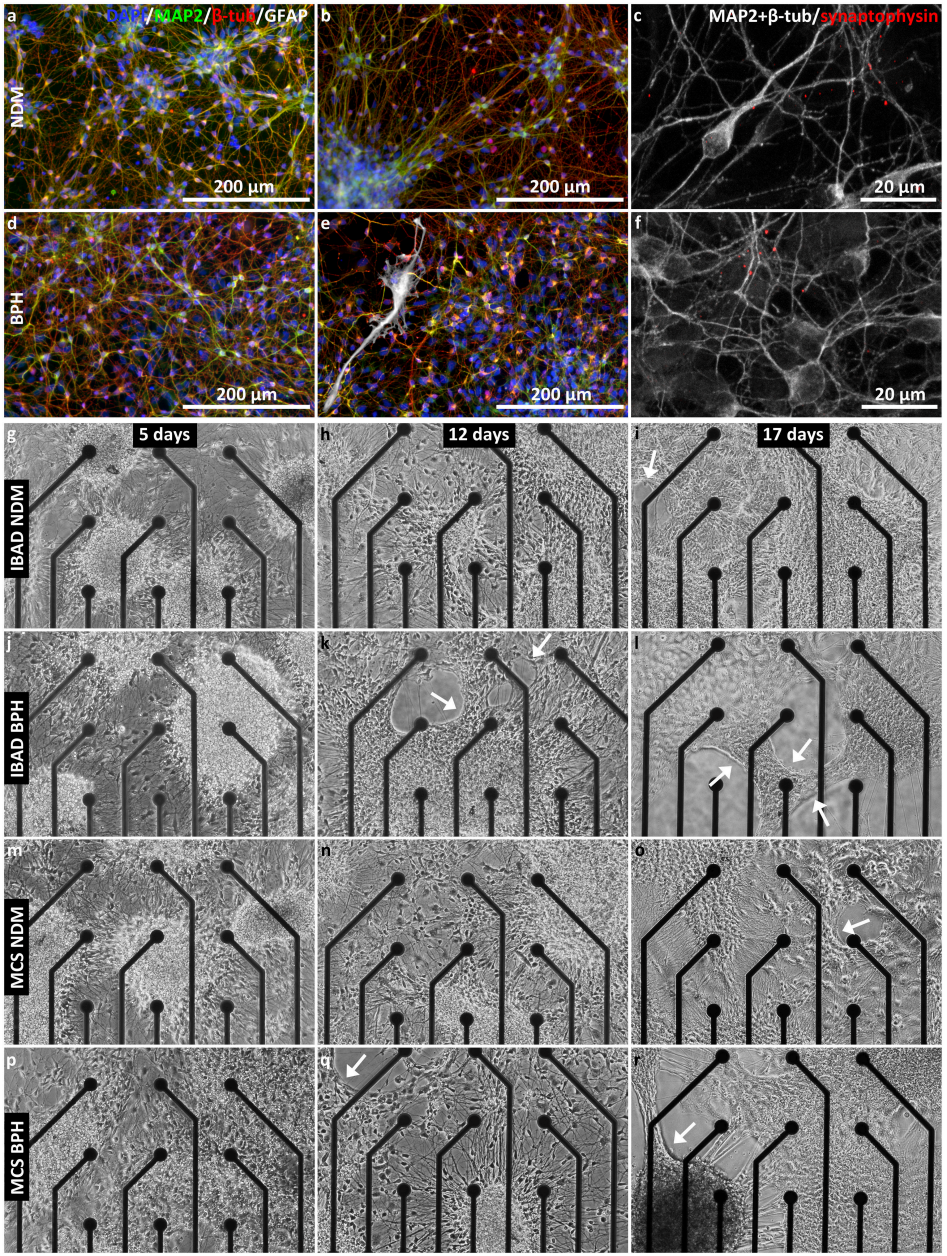
Neuronal networks in NDM and BPH media. **(a–f)** Immunocytochemistry images from neuronal networks in NDM **(a–c)** and BPH **(d–f)** after 12 days in adherent culture. Networks in both media contained MAP2 and β-tub-positive neurons, whereas GFAP-positive astrocytes were only found in BPH medium **(e)**. Synaptophysin was expressed in networks in both media **(c,f)**. **(g–r)** Development of the network in NDM **(g–j,m–o)** and BPH media **(j–l,p–r)** on BMT MEAs **(g–l)** and MCS MEAs **(m–r)** at 5, 12, and 17 days in adherent culture. Network retraction (indicated by arrows) started earlier in BPH **(k** and **q)** than in NDM (**i** and **o**) on both MEA types.

Human pluripotent stem cell (hPSC)-derived neurons were cultured in neural differentiation medium (NDM) and BrainPhys medium (BPH) in control cell culture plastic wells. Both medium types supported the formation of MAP2 and β-tub-positive neuronal networks with expression of synaptophysin (Figures 5a–f) during a 12 days follow-up period. However, GFAP-positive astrocytes were only found in cultures supplemented with BPH medium (Figure 5e). Even though the network formation was good in both media, the organization of the networks differed between them. The neuronal networks were denser in BPH than those in NDM medium (Figures 5c,f). Neuronal cells migrated out of the cell aggregates in both media but more extensively in BPH. In NDM, neuronal cells remaining in the aggregates extended long neurites, which were less common in BPH.

Even though the networks grew well in both media in both MEA types at the beginning of the experiment (Figures 5g,j,m,p), after 1–2 weeks the neuronal networks started to retract and form clumps in BPH (Figures 5k,q). Network retraction also occurred in some NDM wells but typically later than in BPH (Figures 5h,n,i,o vs Figures 5k,q,l,r). The results were the same for both MEA types. The cultures were kept for 19–20 days on MEAs until the network retraction was too extensive, especially in BHP medium, for further measurements. Example videos of network growth on control plates after 10 days in adherent culture (Supplementary videos 1–3) show the typical behavior of the neuronal networks in both media over 26 h. At this point, the networks were not yet retracting in NDM or BPH on cell culture plastic (Supplementary videos 1, 2). However, network retraction in BHP on coverslips was substantial (Supplementary video 3). The cell culture experiments were repeated, and similar results were obtained. Network retraction, clump formation, and cell detachment occurred first and were more prominent in BPH than in NDM.

#### Development of Electrophysiological Activity in NDM and BPH Media on BMT and MCS MEAs

**Figure 6:**
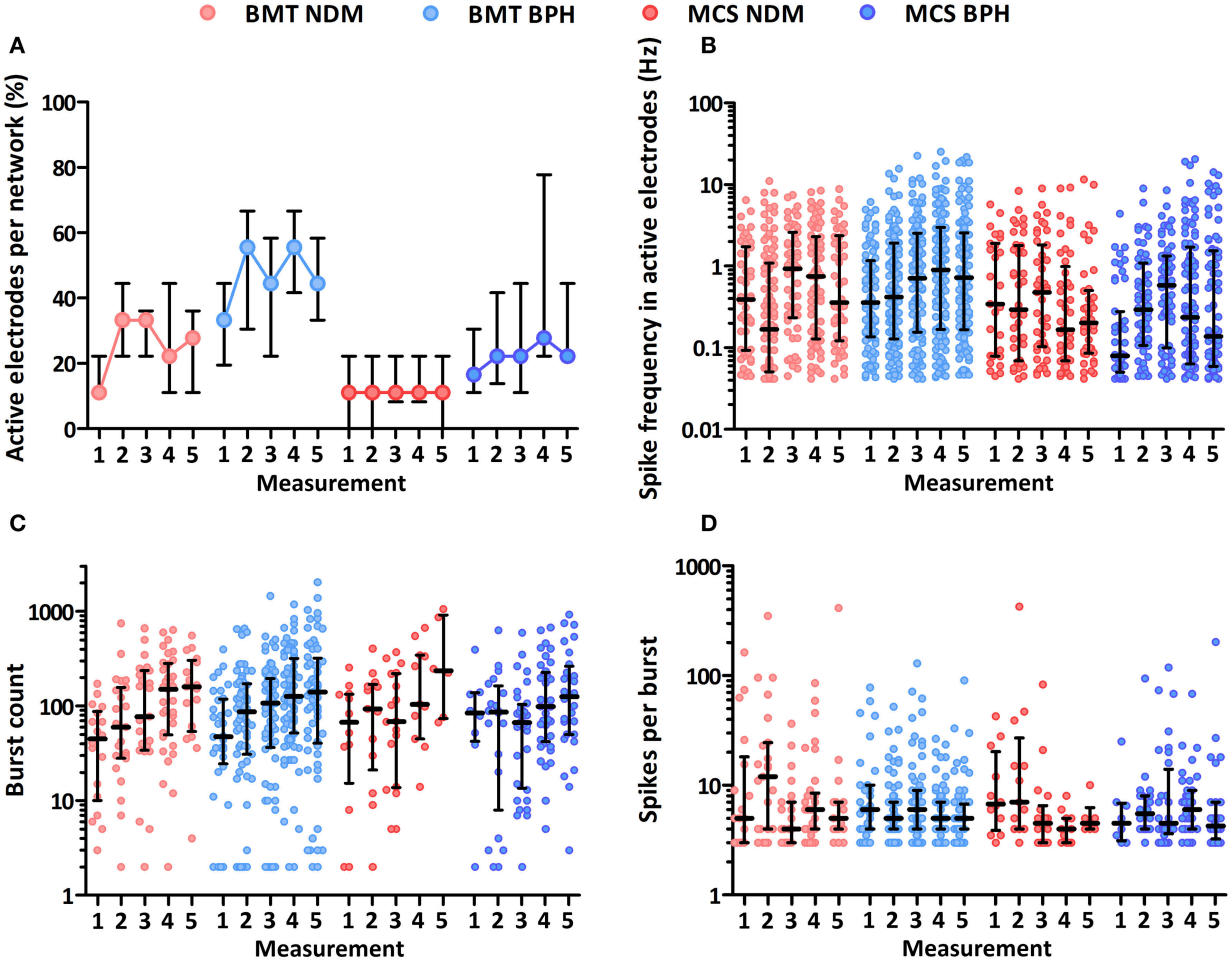
MEA activity from NDM and BPH media on BMT and MCS MEAs. **(A)** Percentage of active electrodes per network (spike frequency >0.04 Hz). Higher percentages of electrodes were active in BPH on both MEA types. **(B)** Median spike frequency in active electrodes. **(C)** Median burst count over 10 min. **(D)** Spikes per burst median. Measurement 1 = 6 days, 2 = 11 days, 3 = 13 days, 4 = 18 days, and 5 = 19–20 days in adherent culture. Median and interquartile ranges are shown.

The percentage of active electrodes (spike frequency >0.04 Hz) per network was higher in BPH than in NDM at all measurement time points (M1 = 6 days, M2 = 11 days, M3 = 13 days, M4 = 18 days, and M5 = 19–20 days) on both BMT and MCS MEAs (Figure 6A). The results were statistically significant at most of the time points (BMT M1 *p* = 0.005, M2 *p* = 0.035, M4 *p* < 0.001; MCS M1 *p* = 0.030, M3 *p* = 0.045, M4 *p* < 0.001, M5 *p* < 0.001). Even though the BPH increased the amount of active electrodes, the median spike frequency in active electrodes was not clearly increased in BPH medium (Figure 6B). Depending on the measurement time point, more spikes were recorded in either BPH or NDM medium in both MEA types. Furthermore, the median burst count during the 10 min recording was not higher in BPH than that in NDM (Figure 6C). However, more electrodes recorded bursts in BPH medium. The spikes per burst medians were rather similar between the media in both MEA types (Figure 6D). Additional MEA analysis results are presented in Supplementary Tables 1, 2. Overall, BPH medium increased the amount of active electrodes but did not enhance the spike frequency or network maturation based on the burst parameters.

#### Insulation Layer Corrosion

**Figure 7:**
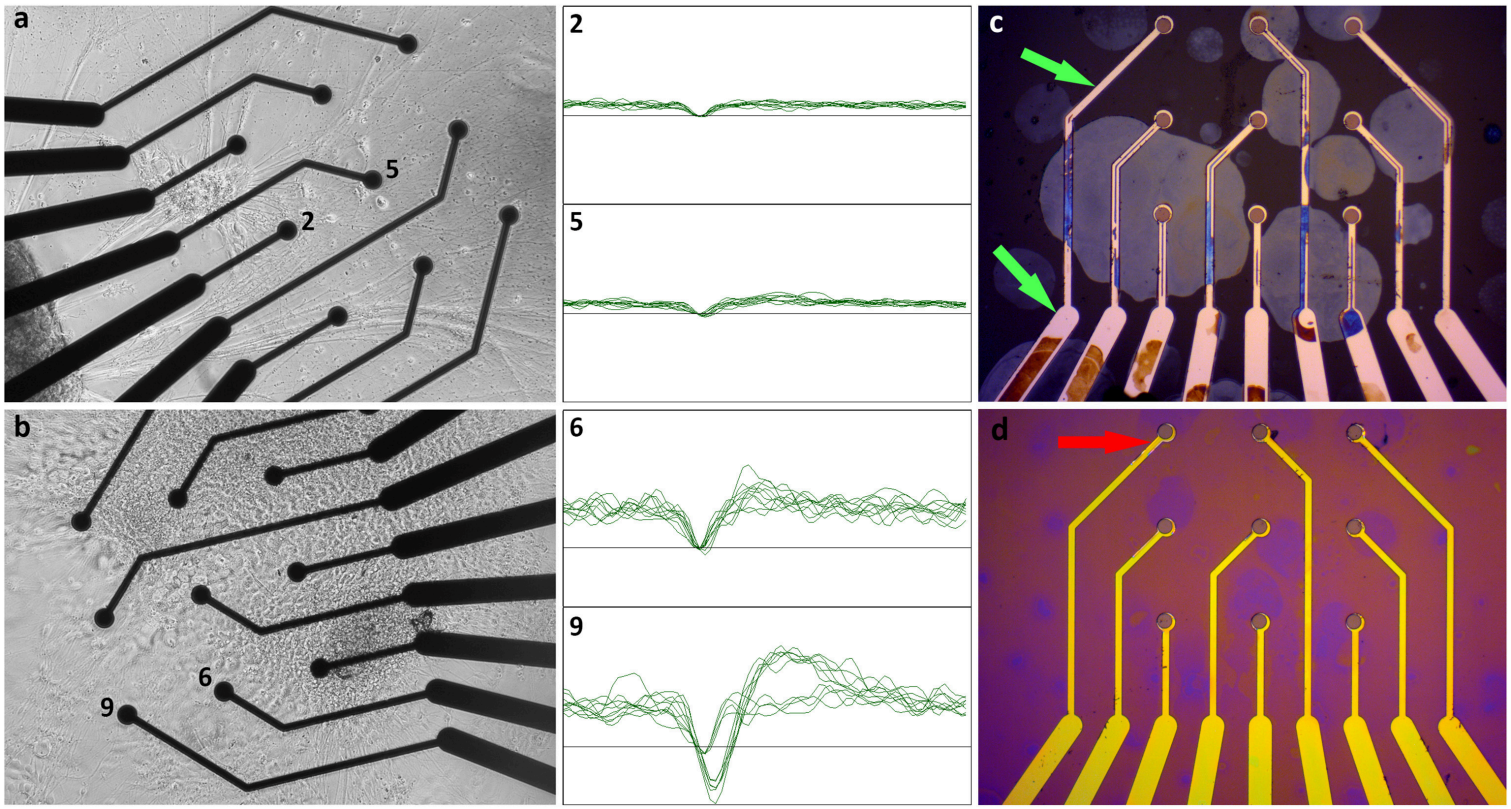
Effect of corroded insulation on signal amplitude. **(a)** Neuronal network in BPH medium on a BMT MEA well with a badly corroded insulation layer after 21 days in adherent culture and corresponding spike waveforms from day 22 recordings from two representative electrodes (2 and 5). **(b)** Neuronal network in NDM medium at 17 days in adherent culture and recorded waveforms (6 and 9) from day 20 on a BMT MEA still with a proper insulation layer. The boxes are ±50 μV and 3 ms. **(c)** Example of insulator layer corrosion in BMT MEA after 3 weeks of cell culture in BPH medium. The green arrows indicate examples of fully corroded areas, demonstrating that most of the tracks have lost their insulation. **(d)** Example of insulator layer corrosion in BMT MEA after 3 weeks of cell culture in NDM medium. The insulator layer may have thinned all around, but it is fully corroded only from a small, barely visible area near the electrode as indicated by the red arrow.

Corrosion of the insulator layer in BMT MEAs not only affect the reliability of the impedance readings after the cell experiments but the vanishing of the insulation also caused a decrease in the MEA signal amplitudes. Examples of spike waveforms recorded using MEA with a badly corroded insulation layer are presented in Figure 7a. In comparison to spike waveforms recorded with MEA still with proper insulation left (Figure 7b), the signal amplitude from the badly corroded MEAs is substantially lower. In addition, the amplitude of the noise was lower in the badly corroded MEAs.

## Discussion

The aim of this study was to demonstrate that sputtering is not the only method for fabricating TiN microelectrodes, as an alternative method exists. For the sputtered commercial TiN microelectrodes (MCS), we measured an impedance range of 30–50 kΩ, which is in line with the range provided in the manufacturer's brochure. This range is < 80 kΩ as reported by Egert et al. (1998) in an early paper describing the sputtered TiN process for MEAs. Thus, it is very likely that by continuing process parameter optimization we could cut some tens of kΩ from the impedance of IBAD TiN microelectrodes. Our results showed that with IBAD TiN we reached impedance levels < 100 kΩ, similar to those of current sputtered TiN microelectrodes. The signal-to-noise ratio was also very similar between these types of MEA electrodes. The CTC values reported in literature for sputtered TiN microelectrodes vary a lot. The original record by Janders et al. (1996) was as high as 42 mC/cm^2^, which was later questioned by Weiland et al. (2002) who reported ~2.4 mC/cm^2^. That is in line with our result for sputtered TiN, 2.0 mC/cm^2^. On the contrary, Gerwig et al. (2012) and Li et al. (2011) have reported both only 0.45 mC/cm^2^. So, if Janders' record is ignored, IBAD TiN seems to perform well against sputtered TiN with its CTC of 3.3 mC/cm^2^ also in this aspect. Thus, IBAD TiN microelectrodes can be expected to be competitive also in stimulation use. The produced IBAD TiN MEAs were compatible for cell measurements, especially when TiN is concerned. Overall, for in-house production, the availability of deposition equipment determines which TiN production method is used. For operators with an e-beam but no sputtering system, upgrading the e-beam coater with an ion source could be the most economical choice to obtain tools for in-house TiN deposition.

Here, the IBAD deposition parameters used in MEA fabrication were chosen based on the SAR; higher SAR was expected to result in lower impedance. Mumtaz and Class (1982) linked brownish color to more porous TiN structure, which agrees with our SAR results and color observations. Further, the coating colors of the produced samples were in line with the observations from previous studies (Mumtaz and Class, 1982; Roquiny et al., 1999). Additionally, it might be interesting to also fabricate MEAs with IBAD deposition parameters other than the ones chosen here as optimal, just to confirm whether the highest SAR is the true defining factor of the lowest impedance. The SAR results here are based on AFM sampling of only two 1 μm × 1 μm areas per sample, which may also leave room for error. However, the measured surface roughness value of 3.1 nm is in good agreement with the value of 3.0 nm by Cyster et al. (2002) for their DC magnetron-sputtered TiN. They also observed a rather strong dependency between the TiN layer thickness and roughness, which is in agreement with our observation of higher thickness meaning lower impedance. In order to keep the MEA surface rather planar and to avoid difficulties in certain process steps there is, however, not much room to play with the TiN thickness. Other parameters commonly connected to IBAD but not evaluated in this study are the substrate temperature and the ion beam incident angle which both may affect on the thin film properties. In our system adjusting those two parameters just was not possible. However, we did observe some temperature increase inside the deposition chamber after the IBAD process, but according to the ion source manufacturer, Saintech, with their ion sources the increase in the substrate temperature should be only very modest 20–30°C compared to ambient temperature, even if the ion source were operated on full power.

When comparing BMT-and MCS-fabricated MEAs, one should also note that there are some minor differences in the design. Only the electrode area of both BMT and MCS MEAs is equal in layout. Wider parts of the tracks and contact pads, on the contrary, have some “artistic” differences as we did not have MCS's mask layout CAD files available. As MCS brochures reveal only the insulator layer thickness, it is possible that Ti and TiN thicknesses are not equal in both MEA types. These differences can probably generally be ignored, but there was a notable difference in the corrosion resistance of the insulator layer even if both fabricators use 500 nm PECVD Si_3_N_4_. As we also observed similar corrosion in a control MEA with no IBAD TiN layer on titanium electrodes, we are confident that the IBAD TiN deposition process itself, despite potential thermal expansion-related issues, or the related rather long ultrasound bath during lift-off, is not the reason for the more corrosion-prone insulator layer in BMT MEAs. Our PECVD process only produces lower quality Si_3_N_4_ compared with MCS's process and requires further optimization. One should note that the IBAD TiN process introduced here is by no means connected to our PECVD Si_3_N_4_ process and whoever adapts IBAD TiN to their MEAs is free to use whatever insulator layer they find the most suitable for their application. If one does not want to move to polymer insulators like polyimide or SU-8, one common solution is to replace Si_3_N_4_ with a more stable but harder to etch sandwich structure of SiO_2_/Si_3_N_4_/SiO_2_ (Buitenweg et al., 1998; Yeung et al., 2007). Even if we did not observe as strong insulator layer corrosion in MCS's MEAs, these MEAs do still present some corrosion, and we have seen outside this study that, in the long run, the MCS MEA insulator layer does eventually totally wear out as well. Also Wagenaar et al. (2004) have reported long term insulator failure in MCS MEAs. In fact, there are more common studies (Schmitt et al., 2000; Herrera Morales, 2015), where not only Si_3_N_4_ but also many other commonly used insulator materials have been found to have a poor corrosion resistance in a biological environment. It is evident that the MEA community should stop focusing only on developing new electrode materials and put effort on studying the insulator materials as well.

Another interesting finding related to TiN was that the impedance of both sputtered and IBAD-deposited microelectrodes increased greatly during the experiments. As the impedance also increased for the control group of IBAD TiN MEAs not subject to any cell experiments, it is not necessarily only the cells or cell culture medium that either harms the electrode material or leaves some type of impedance-increasing residual on the surface of the MEA. It seems that storing the MEAs in normal room atmosphere between experiments may be the primary reason for electrode degradation, likely due to the partial oxidation of Ti(N). In our preliminary tests of storing IBAD TiN MEAs in deionized water in a refrigerator, the electrodes retained constant impedance for a 1 week test period. An open question is whether the increasing impedance saturates at some point and the performance of the MEA at this point. As the condition of MEA is often controlled by checking the impedance, the combination of insulator layer corrosion and increasing electrode impedance due to oxidation makes the evaluation of the condition of MEA somewhat tricky; the impedance may stay within certain limits not because of MEA being consistent but because those two factors have compensated for each other.

In the cell culture experiments, we tested 2 media with both type of MEAs. In BPH, a significant increase in active electrode percentages was found compared to NDM. Earlier, similar results have been shown for BPH compared to standard cell culture medium (Bardy et al., 2015). Overall, our results may indicate that BPH enhanced the activity of the neurons directly or enabled denser neuronal network organization compared to NDM (see all the details in Supplementary Table 1). Interestingly, BPH did not increase the spike frequencies in this study in contrast to reported by Bardy et al. (2015). The burst count was neither increased in BPH compared to NDM, thus showing in both media the typical developmental increase during 3 weeks follow up as previously reported (Heikkilä et al., 2009). The median spikes per a burst (Figure 6D) as well as other burst parameters (Supplementary Table 2) showed no statistical differences between BPH and NDM. These results indicate that BPH did not enhance the maturation of the neuronal networks.

Importantly, our results revealed that BPH did not support long-term cell culturing in either of MEA types as neuronal networks started to retract and form cell clumps after 1–2 weeks and resulted in experiment termination in 3 weeks timepoint. Network retraction and cell clumping has been mentioned also in the study of Bardy et al. (2015) as a minor problem while here in our longer study it became a major problem. The long term stability of the network is most important for hPSC-derived neurons that require several weeks or even longer to develop mature neuronal activity (Heikkilä et al., 2009; Odawara et al., 2016).

In summary, we verified that IBAD is a valid method for producing TiN electrodes for MEA systems. Thus, it can be considered as an alternative TiN deposition method for sputtering. We also stated that BPH medium supported the development of neuronal activity on MEAs, although it caused problems in cell behavior and MEA insulator layer stability in long-term cultures. Thus, as insulator material, electrode material, and even cell culture medium can have detrimental effects on recording quality of MEAs especially with long-term cultures, all of these aspects should be carefully evaluated.

## Author Contributions

TR is responsible for the IBAD TiN process development, BMT MEA design and fabrication, AFM, impedance, and CV measurements, and technical data analysis excluding noise analysis performed together with MT and LY-O. MT is responsible for the cell experiments and cell data analysis. TS operated SEM and EDS. TR and MT wrote the manuscript. LY-O, SN, and JL participated in the project design with TR and MT and provided additional support to analysis and writing of the manuscript.

### Conflict of Interest Statement

The authors declare that the research was conducted in the absence of any commercial or financial relationships that could be construed as a potential conflict of interest.

## References

[B1] Bardy C. van den Hurk M. Eames T. Marchand C. Hernandez R. V. Kellogg M.. (2015). Neuronal medium that supports basic synaptic functions and activity of human neurons *in vitro*. PNAS 112, E2725–E2734. 10.1073/pnas.1504393112 25870293 PMC4443325

[B2] Bauerdick S. Burkhardt C. Kern D. P. Nisch W. (2003). BioMEMs materials and fabrication technology : substrate-integrated microelectrodes with improved charge transfer capacity by 3-dimensional micro-fabrication. Biomed. Microdevices 5, 93–99. 10.1023/A:1024526626016

[B3] Buitenweg J. R. Rutten W. L. Willems W. P. van Nieuwkasteele J. W. (1998). Measurement of sealing resistance of cell-electrode interfaces in neuronal cultures using impedance spectroscopy. Med. Biol. Eng. Comput. 36, 630–637. 10.1007/BF02524436 10367450

[B4] Cogan S. F. (2008). Neural stimulation and recording electrodes. Annu. Rev. Biomed. Eng. 10, 275–309. 10.1146/annurev.bioeng.10.061807.160518 18429704

[B5] Cyster L. A. Grant D. M. Parker K. G. Parker T. L. (2002). The effect of surface chemistry and structure of titanium nitride (TiN) films on primary hippocampal cells. Biomol. Eng. 19, 171–175. 10.1016/S1389-0344(02)00021-7 12202178

[B6] Egert U. Schlosshauer B. Fennrich S. Nisch W. Fejtl M. Knott T.. (1998). A novel organotypic long-term culture of the rat hippocampus on substrate-integrated multielectrode arrays. Brain Res. Brain Res. Protoc. 2, 229–242. 10.1016/S1385-299X(98)00013-0 9630647

[B7] Falk A. Heine V. Harwood A. PF S. Peitz M. Brüstle O. (2016). Modeling psychiatric disorders: from genomic findings to cellular phenotypes. Mol. Pychiatry 21, 1167–1179. 10.1038/mp.2016.89 PMC4995546 27240529

[B8] Gabay T. Ben-David M. Kalifa I. Sorkin R. Abrams Z. R. Ben-Jacob E.. (2007). Electro-chemical and biological properties of carbon nanotube based multi-electrode arrays. Nanotechnology 18:035201. 10.1088/0957-4484/18/3/035201 19636111

[B9] Gahlin R. Bromark M. Hedenqvist P. Hogmark S. Hakansson G. (1995). Properties of TiN and CrN coatings deposited at low temperature using reactive arc-evaporation. Surf. Coatings Technol. 77, 174–180. 10.1016/0257-8972(95)02597-9

[B10] Gawad S. Giugliano M. Heuschkel M. Wessling B. Markram H. Schnakenberg U.. (2009). Substrate arrays of iridium oxide microelectrodes for *in vitro* neuronal interfacing. Front. Neuroeng. 2:1. 10.3389/neuro.16.001.2009 19194527 PMC2634525

[B11] Gerwig R. Fuchsberger K. Schroeppel B. Link G. S. Heusel G. Kraushaar U.. (2012). PEDOT–CNT composite microelectrodes for recording and electrostimulation applications: fabrication, morphology, and electrical properties. Front. Neuroeng. 5:8. 10.3389/fneng.2012.00008 22586394 PMC3343311

[B12] Guzman L. Bonelli M. Miotello A. Kothari D. C. (1998). Process parameters optimization for TiN and TiC formation using reactive ion beam assisted deposition. Surf. Coatings Technol. 100–101, 500–502. 10.1016/S0257-8972(97)00679-8

[B13] Heikkilä T. J. Ylä-Outinen L. Tanskanen J. M. A. Lappalainen R. S. Skottman H. Suuronen R.. (2009). Human embryonic stem cell-derived neuronal cells form spontaneously active neuronal networks *in vitro*. Exp. Neurol. 218, 109–116. 10.1016/j.expneurol.2009.04.011 19393237

[B14] Heim M. Rousseau L. Reculusa S. Urbanova V. Mazzocco C. Joucla S.. (2012). Combined macro-/mesoporous microelectrode arrays for low-noise extracellular recording of neural networks. J. Neurophysiol. 108, 1793–1803. 10.1152/jn.00711.2011 22745460

[B15] Herrera Morales J. M. (2015). Selecting and Evaluating Biocompatible Barrier Films for Protecting Medical Micro Devices. Dissertation. Université Grenoble Alpes.

[B16] Huang J. Lin C. Ma C. Chen H. (2000). Low energy ion beam assisted deposition of TiN thin films on silicon. Scr. Mater. 42, 573–579. 10.1016/S1359-6462(99)00393-0

[B17] Hubler G. K. Vanvechten D. Donovan E. P. Kant R. A. (1988). Ion beam assisted deposition of titanium nitride. MRS Proc. 128, 55–60. 10.1557/PROC-128-55

[B18] Janders M. Egert U. Stelzle M. Nisch W. (1996). “Novel thin film titanium nitride micro-electrodes with excellent charge transfer capability for cell stimulation and sensing applications,” in Proceedings of 18th Annual International Conference of the IEEE Engineering in Medicine and Biology Society (1996) Amsterdam, 245–247.

[B19] Jiang N. Zhang H. J. Bao S. N. Shen Y. G. Zhou Z. F. (2004). XPS study for reactively sputtered titanium nitride thin films deposited under different substrate bias. Phys. B Condens. Matter 352, 118–126. 10.1016/j.physb.2004.07.001

[B20] Johnstone A. F. Gross G. W. Weiss D. G. Schroeder O. H. Gramowski A. Shafer T. J. (2010). Microelectrode arrays: a physiologically based neurotoxicity testing platform for the 21st century. Neurotoxicology 31, 331–350. 10.1016/j.neuro.2010.04.001 20399226

[B21] Kapucu F. E. Tanskanen J. M. Mikkonen J. E. Ylä-Outinen L. Narkilahti S. Hyttinen J. A. (2012). Burst analysis tool for developing neuronal networks exhibiting highly varying action potential dynamics. Front. Comput. Neurosci. 6:38. 10.3389/fncom.2012.00038 22723778 PMC3378047

[B22] Kreutzer J. Ylä-Outinen L. Kärnä P. Kaarela T. Mikkonen J. Skottman H. (2012). Structured PDMS chambers for enhanced human neuronal cell activity on MEA platforms. J. Bionic Eng. 9, 1–10. 10.1016/S1672-6529(11)60091-7

[B23] Lappalainen R. S. Salomäki M. Ylä-Outinen L. Heikkilä T. J. Hyttinen J. A. Pihlajamäki H.. (2010). Similarly derived and cultured hESC lines show variation in their developmental potential towards neuronal cells in long-term culture. Regen. Med. 5, 749–762. 10.2217/rme.10.58 20868330

[B24] Li X. Pei W. Tang R. Gui Q. Guo K. Wang Y. (2011). Investigation of flexible electrodes modified by TiN, Pt black and IrO x. Sci. China Technol. Sci. 54, 2305–2309. 10.1007/s11431-011-4436-7

[B25] López J. M. Gordillo-Vázquez F. J. Böhme O. Albella J. M. (2001). Low grain size TiN thin films obtained by low energy ion beam assisted deposition. Appl. Surf. Sci. 173, 290–295. 10.1016/S0169-4332(00)00912-0

[B26] Mumtaz A. Class W. H. (1982). Color of titanium nitride prepared by reactive dc magnetron sputtering. J. Vac. Sci. Technol. 20, 345–348. 10.1116/1.571461

[B27] Odawara A. Katoh H. Matsuda N. Suzuki I. (2016). Physiological maturation and drug responses of human induced pluripotent stem cell-derived cortical neuronal networks in long-term culture. Sci. Rep. 6:26181. 10.1038/srep26181 27188845 PMC4870631

[B28] Peng H. Zhou D. Zhang J. Guo H. Gong S. (2015). Deposition of TiN by plasma activated EB-PVD: activation by thermal electron emission from molten niobium. Surf. Coatings Technol. 276, 645–648. 10.1016/j.surfcoat.2015.05.047

[B29] Quiroga R. Q. Nadasdy Z. Ben-Shaul Y. (2004). Unsupervised spike detection and sorting with wavelets and superparamagnetic clustering. Neural Comput. 16, 1661–1687. 10.1162/089976604774201631 15228749

[B30] Roquiny P. Bodart F. Terwagne G. (1999). Colour control of titanium nitride coatings produced by reactive magnetron sputtering at temperature less than 100??C. Surf. Coatings Technol. 116–119, 278–283. 10.1016/S0257-8972(99)00076-6

[B31] Ryynänen T. Toivanen M. Narkilahti S. Lekkala J. (2016). Titanium Nitride Microelectrodes Deposited by Ion Beam Assisted E-beam Evaporation. Front. Neurosci. Conference Abstract: MEA Meeting 2016 | 10th International Meeting on Substrate-Integrated Electrode Arrays. 10.3389/conf.fnins.2016.93.00123

[B32] Samba R. Fuchsberger K. Matiychyn I. Epple S. Kiesel L. Stett A. (2014). Application of PEDOT-CNT microelectrodes for neurotransmitter sensing. Electroanalysis 26, 548–555. 10.1002/elan.201300547

[B33] Schmitt G. Faßbender F. Lüth H. Schöning M. J. Schultze J.-W. Buß, G. (2000). Passivation and corrosion of microelectrode arrays. Mater. Corr. 51, 20–25. 10.1002/(SICI)1521-4176(200001)51:1<20::AID-MACO20>3.0.CO;2-Q

[B34] Stelzle M. Stett A. Brunner B. Graf M. Nisch W. (2001). Electrical properties of micro-photodiode arrays for use as artificial retina implant. Biomed. Microdevices 3, 133–142. 10.1023/A:1011450326476

[B35] Thomas C. A. Springer P. A. Loeb G. E. Berwald-Netter Y. Okun L. M. (1972). A miniature microelectrode array to monitor the bioelectric activity of cultured cells. Exp. Cell Res. 74, 61–66. 10.1016/0014-4827(72)90481-8 4672477

[B36] Wagenaar D. A. Pine J. Potter S. M. (2004). Effective parameters for stimulation of dissociated cultures using multi-electrode arrays. J. Neurosci. Methods 138, 27–37. 10.1016/j.jneumeth.2004.03.005 15325108

[B37] Wagner J. Mitterer C. Penoy M. Michotte C. Wallgram W. Kathrein M. (2008). The effect of deposition temperature on microstructure and properties of thermal CVD TiN coatings. Int. J. Refract. Met. Hard Mater. 26, 120–126. 10.1016/j.ijrmhm.2007.01.010

[B38] Weiland J. D. Anderson D. J. Humayun M. S. (2002). *In vitro* electrical properties for iridium oxide versus titanium nitride stimulating electrodes. IEEE Trans. Biomed. Eng. 49, 1574–1579. 10.1109/TBME.2002.805487 12549739

[B39] Xie S. Cai J. Wang Q. Wang L. Liu Z. (2014). Properties and morphology of TiN films deposited by atomic layer deposition. Tsinghua Sci. Technol. 19, 144–149. 10.1109/TST.2014.6787367

[B40] Yeung C. K. Sommerhage F. Wrobel G. Offenhäusser A Chan M. Ingebrandt S. (2007). Drug profiling using planar microelectrode arrays. Anal. Bioanal. Chem. 387, 2673–2680. 10.1007/s00216-007-1172-8 17318515

[B41] Ylä-Outinen L. Heikkilä J. Skottman H. Suuronen R. Aänismaa R. Narkilahti S. (2010). Human cell-based micro electrode array platform for studying neurotoxicity. Front. Neuroeng. 3:111. 10.3389/fneng.2010.00111 20953240 PMC2955435

[B42] Yokota K. Nakamura K. Kasuya T. Mukai K. Ohnishi M. (2004). Resistivities of titanium nitride films prepared onto silicon by an ion beam assisted deposition method. J. Phys. D. Appl. Phys. 37, 1095–1101. 10.1088/0022-3727/37/7/023

