# Supplementary material for: Ion Beam Assisted E-Beam Deposited TiN Microelectrodes—Applied to Neuronal Cell Culture Medium Evaluation
Source: Front Neurosci. 2018 Dec 4;12:882. doi: 10.3389/fnins.2018.00882 (PMC6290344; doi:10.3389/fnins.2018.00882)
Supplement: Supplementary file 1 [file Table_1.pdf]

![frontiers logo]

Supplementary Material

Ion beam assisted e-beam deposited TiN microelectrodes – applied to neuronal cell culture medium evaluation

Tomi Ryynänen*, Maria Toivanen, Turkka Salminen, Laura Ylä-Outinen, Susanna Narkilahti, Jukka Lekkala

*** Correspondence:** Tomi Ryynänen: tomi.ryynanen@tut.fi

1 Supplementary Tables

Table 1 | Additional MEA analysis results of spikes and bursts on each MEA type and medium combination. Time points: M1 = 6 days, M2 = 11 days, M3 = 13 days, M4 = 18 days, and M5 = 19-20 days.

Time point and MEA type	Medium	Median	Range	Mean	SD	p	Median	Range	Mean	SD	p	Median	Range	Mean	SD	p
		Median spike frequency in burst (Hz)					Median spikes per burst					Median spike frequency in burst (Hz)				
M1	NDM	7	70	19	21	0,374	820	56616	6259	14229	0,531	5	160	22	41	0,611
BMT	BPH	10	681	91	205		665	14912	2448	3962		6	75	13	18	
M2	NDM	8	748	57	163	0,064	1588	46735	6598	11259	0,010	12	347	36	74	0,031
BMT	BPH	13	748	89	195		386	27622	1397	3861		5	49	8	8	
M3	NDM	17	796	97	223	0,779	188	10596	1218	2510	0,138	4	34	7	8	0,057
BMT	BPH	16	609	69	161		403	42855	3766	9824		6	126	12	20	
M4	NDM	14	361	30	65	0,085	402	52329	3193	9304	0,087	6	83	12	18	0,100
BMT	BPH	19	997	51	155		265	3573	515	679		5	24	6	4	
M5	NDM	12	68	18	17	0,001	472	141334	8995	34130	0,012	5	411	30	99	0,577
BMT	BPH	31	998	72	161		189	41494	1124	5275		5	88	8	12	
M1	NDM	7	581	86	191	0,208	1774	10517	2962	3610	0,238	7	40	12	12	0,208
MCS	BPH	17	351	68	121		441	2803	859	1014		5	22	7	7	
M2	NDM	6	114	16	31	0,020	1921	295612	26903	81151	0,087	7	423	45	115	0,265
MCS	BPH	12	731	59	162		470	6354	1053	1784		6	91	10	20	
M3	NDM	11	105	24	28	0,887	452	33559	2646	7907	0,558	5	80	10	19	0,344
MCS	BPH	14	315	26	55		432	55976	4131	11474		5	116	15	25	
M4	NDM	15	40	19	13	0,371	294	1124	358	315	0,397	4	5	4	2	0,008
MCS	BPH	18	998	49	162		319	4175	711	1066		6	65	9	11	
M5	NDM	11	41	17	15	0,145	354	2073	613	773	0,413	5	6	5	2	0,809
MCS	BPH	25	85	33	26		206	21884	1595	4559		4	200	14	38	

NDM = Neuronal differentiation Medium, BHP = BrainPhys medium, BMT = BMT produced IBAD-TiN MEAs, MCS = MEAs from Multi Channel Systems GmbH, SD = standard deviation, p = p-value calculated pairwise between NMD and BPH using Mann-Whitney U-test ($p \leq 0.05$ is considered as significant and marked as yellow).

Table 2 | Additional MEA analysis results of active and burst detecting electrodes on each MEA type and medium combination. Time points: M1 = 6 days, M2 = 11 days, M3 = 13 days, M4 = 18 days, and M5 = 19-20 days.

Time point and MEA type	Medium	Median	Range	Mean	SD	p	Median	Range	Mean	SD	p
		Active electrodes/ network (%)					Spike frequency in active electrodes (Hz)				
M1	NDM	11	56	17	13	0.001	0.389	6.377	1.045	1.401	0.988
BMT	BPH	33	67	31	17		0.360	6.054	0.963	1.300	
M2	NDM	33	78	34	18	0.007	0.168	11.028	1.035	1.972	0.001
BMT	BPH	56	78	51	23		0.418	15.648	1.395	2.394	
M3	NDM	33	56	31	16	0.068	0.933	7.415	1.575	1.849	0.938
BMT	BPH	44	78	43	22		0.712	22.358	1.940	3.088	
M4	NDM	22	89	27	22	<0.001	0.748	8.375	1.592	1.998	0.243
BMT	BPH	56	89	50	22		0.898	25.207	2.462	3.823	
M5	NDM	28	67	28	19	0.011	0.358	8.742	1.474	1.964	0.265
BMT	BPH	44	89	44	22		0.720	21.703	2.483	4.292	
M1	NDM	11	33	10	10	0.006	0.342	5.664	1.172	1.476	0.002
MCS	BPH	17	67	22	17		0.080	4.327	0.399	0.751	
M2	NDM	11	33	14	12	0.020	0.293	8.338	1.227	1.773	0.683
MCS	BPH	22	67	26	19		0.295	8.914	0.901	1.550	
M3	NDM	11	44	16	13	0.009	0.477	8.890	1.237	1.853	0.777
MCS	BPH	22	78	29	20		0.583	8.480	1.092	1.489	
M4	NDM	11	44	15	12	<0.001	0.167	9.163	1.064	2.094	0.648
MCS	BPH	28	89	43	30		0.235	20.448	1.782	3.722	
M5	NDM	11	44	14	14	<0.001	0.202	11.468	1.031	2.447	0.705
MCS	BPH	22	89	35	24		0.138	14.178	1.676	3.181	
		Burst detecting electrodes/network (%)					Total bursts in burst detecting electrodes/10 min				
M1	NDM	11	22	7	7	0,141	45	169	55	49	0,498
BMT	BPH	11	56	11	13		48	395	83	89	
M2	NDM	0	56	9	13	<0,001	60	746	116	162	0,576
BMT	BPH	22	67	25	19		87	657	127	147	
M3	NDM	11	33	14	11	0,024	77	662	156	168	0,853
BMT	BPH	22	56	24	15		107	1456	156	210	
M4	NDM	11	44	12	13	<0,001	151	635	186	170	0,953
BMT	BPH	22	67	30	17		127	1179	203	214	
M5	NDM	6	44	10	13	0,009	160	552	193	154	0,835

BMT	BPH	22	56	24	17		141	2031	268	365	
M1	NDM	0	22	4	7	0,716	68	253	86	78	0,624
MCS	BPH	0	22	4	6		84	391	116	121	
M2	NDM	0	22	5	7	0,091	93	402	119	112	0,650
MCS	BPH	11	44	9	11		87	628	115	145	
M3	NDM	0	33	7	9	0,007	69	365	118	117	0,585
MCS	BPH	11	56	15	13		67	593	97	121	
M4	NDM	0	22	4	7	0,004	104	659	231	223	0,447
MCS	BPH	11	67	17	20		99	670	170	169	
M5	NDM	0	22	2	5	0,001	236	989	422	427	0,173
MCS	BPH	11	44	13	14		126	918	216	247	

NDM = Neuronal differentiation Medium, BHP = BrainPhys medium, BMT = BMT produced IBAD-TiN MEAs, MCS = MEAs from Multi Channel Systems GmbH, SD = standard deviation, p= p-value calculated pairwise between NMD and BPH using Mann-Whitney U-test ($p \leq 0.05$ is considered as significant and marked as yellow).

2 Supplementary videos

Video 1 | Example of network growth on a control cell culture plastic plate for over 26 hours after 10 days in adherent culture in NDM medium.

Video1.avi

Video 2 | Example of network growth on a control cell culture plastic plate for over 26 hours after 10 days in adherent culture in BPH medium.

Video2.avi

Video 3 | Example of network growth on a control cover slip for over 26 hours after 10 days in adherent culture in BPH medium.

Video3.avi
